# Chloroquine Causes Aging-like Changes in Diaphragm Neuromuscular Junction Morphology in Mice

**DOI:** 10.3390/cells14060390

**Published:** 2025-03-07

**Authors:** Chloe I. Gulbronson, Sepideh Jahanian, Heather M. Gransee, Gary C. Sieck, Carlos B. Mantilla

**Affiliations:** 1 Department of Anesthesiology & Perioperative Medicine, Mayo Clinic, 200 First Street SW, Rochester, MN 55905, USA; chloe.gulbronson@gmail.com (C.I.G.); jahanian.sepideh@mayo.edu (S.J.); gransee.heather2@mayo.edu (H.M.G.); sieck.gary@mayo.edu (G.C.S.); 2 Department of Physiology & Biomedical Engineering, Mayo Clinic, 200 First Street SW, Rochester, MN 55905, USA

**Keywords:** autophagy, diaphragm muscle, neuromuscular junction, pre-synaptic terminal, motor end-plate

## Abstract

Autophagy impairments have been implicated in various aging conditions. Previous studies in cervical motor neurons show an age-dependent increase in the key autophagy proteins LC3 and p62, reflecting autophagy impairment and autophagosome accumulation. Chloroquine is commonly used to inhibit autophagy by preventing autophagosome–lysosome fusion and may thus emulate the effects of aging on the neuromuscular system. Indeed, acute chloroquine administration in old mice decreases maximal transdiaphragmatic pressure generation, consistent with aging effects. We hypothesized that chloroquine alters diaphragm muscle neuromuscular junction (NMJ) morphology and increases denervation. Adult male and female C57BL/6 × 129J mice between 5 and 8 months of age were used to examine diaphragm muscle NMJ morphology and denervation following daily intraperitoneal injections of chloroquine (10 mg/kg/d) or vehicle for 7 days. The motor end-plates and pre-synaptic terminals were fluorescently labeled with α-bungarotoxin and anti-synaptophysin, respectively. Confocal microscopy was used to assess pre- and post-synaptic morphology and denervation. At diaphragm NMJs, chloroquine treatment decreased pre-synaptic volume by 12% compared to the vehicle (*p* < 0.05), with no change in post-synaptic volume. Chloroquine treatment increased the proportion of partially denervated NMJs by 2.7-fold compared to vehicle treatment (*p* < 0.05). The morphological changes observed were similar to those previously reported in the diaphragm muscles of 18-month-old mice. These findings highlight the importance of autophagy in the maintenance of the structural properties at adult NMJs in vivo.

## 1. Introduction

Chloroquine is a Food and Drug Administration-approved therapeutic prescribed for the treatment of malaria, amebiasis, and various autoimmune diseases [1]. Despite its beneficial effects, chloroquine at therapeutic doses has been associated with many adverse effects, including neuromyopathy, leading to progressive weakness and atrophy of muscle groups [2,3,4]. Numerous studies have implicated chloroquine treatment in neuromuscular dysfunction, including a decrease in neuromuscular transmission [5,6], diaphragm contractility [7], and maximal transdiaphragmatic pressure generation [8]. Although chloroquine’s mechanism of action is not fully understood, chloroquine inhibits autophagy by preventing the autophagosome from fusing with the lysosome, causing the accumulation of autophagosomes [9].

Autophagy is a highly conserved cellular process essential for maintaining cellular homeostasis through the degradation and recycling of damaged cellular components, contributing to overall organismal health [10]. This process comprises the selective targeting of damaged organelles and misfolded proteins, leading to their encapsulation in a double-membraned autophagosome, which is then transported to the lysosome for degradation [11]. This tightly regulated process is implicated in several pathologies that result in cellular dysfunction and degeneration, including aging [12,13]. Many novel compounds have been developed for use in autophagy modulation; however, chloroquine and hydroxychloroquine, a chloroquine-derivative, remain the only autophagy inhibitors approved by the Food and Drug Administration [14]. In the present study, we investigated the effect of chloroquine on denervation of the neuromuscular system by studying the diaphragm muscle, a well-characterized model of aging effects. We hypothesize that chloroquine alters diaphragm muscle neuromuscular junction (NMJ) morphology and increases denervation.

## 2. Materials and Methods

### 2.1. Animals

Adult male (n = 6) and female (n = 6) C57BL/6 × 129J mice between 5 and 8 months of age were examined in our experiments. This age was selected since it precedes the occurrence of age-related changes in autophagy protein levels [12,13]. The mice were group-housed by sex until randomization into treatment groups and housed individually thereafter. All mice were maintained on a 12 h light–dark schedule in specific pathogen-free rooms with free access to food and water throughout their lifespan. All protocols and animal care guidelines were approved by the Institutional Animal Care and Use Committee at the Mayo Clinic (protocol A00003349, approved 5 October 2023), in compliance with National Institute of Health Guidelines.

### 2.2. Experimental Treatment

The mice were randomized to receive intraperitoneal chloroquine (n = 6; 10 mg/kg/day; C6628, Sigma-Aldrich, St. Louis, MO, USA) or vehicle treatment (n = 6; 0.9% saline) for 7 days [15,16]. Equal numbers of male and female animals were allocated to each group. During the terminal experiment, all animals were anesthetized with intraperitoneal injection of ketamine (90 mg/kg) and xylazine (10 mg/kg) and euthanized by exsanguination. The entire diaphragm muscle was dissected and excised for further histologic labeling.

### 2.3. Neuromuscular Junction Morphology

Directly following dissection, the diaphragm muscle was labeled as a whole tissue for morphological assessment of the pre- and post-synaptic structures of the NMJ, as previously described [17,18]. In brief, the diaphragm muscle was pinned on a silicon rubber (Sylgard; Dow-Corning, Midland, MI, USA)-coated dish and fixed in fresh 4% paraformaldehyde. The motor end-plates were labeled via incubation with Alexa 488-conjugated α-bungarotoxin (0.1 µg/mL; B13422, Thermo Fisher Scientific, Waltham, MA, USA) through its binding to cholinergic receptors on the motor end-plate of the muscle fiber [19,20]. Pre-synaptic terminals were labeled by anti-synaptophysin (1:750; 101-004, Synaptic Systems, Gottingen, Germany). An Alexa Fluor 594-conjugated donkey anti-guinea pig IgG secondary antibody was used (1:200; 706-585-148, Jackson ImmunoResearch Labs, West Grove, PA, USA). All diaphragm muscles were stored in Tris-buffered saline at 4 °C until confocal imaging, which occurred no more than 4 days following the staining procedure.

### 2.4. Confocal Imaging and Analysis

The use of confocal microscopy for imaging of NMJs in whole-mount diaphragm muscle has been previously described [17,18,21,22,23]. All confocal imaging and analyses were performed in a blinded manner, such that the treatment group was not known until after all analyses were complete. The diaphragm muscle in whole mount was scanned at low magnification using an Olympus FluoView 300 laser scanning confocal microscope (Evident Scientific, Inc., Waltham, MA, USA) mounted on an upright Olympus BX50WI microscope and equipped with Argon (488 nm) and HeNe (543 nm) lasers to identify areas containing superficial NMJs made visible by fluorescently conjugated α-bungarotoxin (Alexa 488). Images were obtained for all areas containing superficial NMJs at higher magnification using an Olympus LUMPlanFl 40x/0.80 N.A. water immersion lens. Images were acquired in an 800 × 600 array with pixel dimensions (0.5 × 0.5 μm) above the optical resolution for this lens (thus avoiding oversampling). Confocal image stacks were acquired with a 1 μm step size. Selected NMJs were superficial (no more than 60 µm deep) on the thoracic side of the diaphragm muscle to minimize the possible effects of antibody penetration and non-overlapping. At least 35 en face NMJs per animal were identified through α-bungarotoxin labeling.

Analysis of the pre- and post-synaptic structures of diaphragm NMJs were conducted using confocal image stacks consisting of 10–18 optical slices (each with a two-channel 12-bit multi-TIFF file) using MetaMorph (Molecular Devices, Sunnyvale, CA, USA), as previously reported [17,18,23]. A 2D planar area of the motor end-plate was obtained from a maximum-intensity projection, and the planar area was divided by the area described by the main orthogonal axes of the end-plate to calculate the relative planar area [17,18]. The relative planar area reflects the complexity of the motor end-plate indicated by increased branching and fragmentation. A higher relative planar area reflects lower complexity. Volumes of pre- and post-synaptic structures were determined using a customized 3D algorithm in MetaMorph, utilizing manual thresholding as previously described [18]. The intersection of the two binarized volumes was used to determine the volume of apposition, which is expressed as the percentage of the pre-synaptic volume opposing the motor end-plate. Denervation was also qualitatively assessed at individual diaphragm NMJs by determining if there was either complete (innervated), partial (partially denervated), or no overlap (denervated) between pre- and post-synaptic structures based on the maximum intensity projection image.

### 2.5. Statistics

All data were analyzed using JMP (version 17 SAS Institute, Inc., Cary, NC, USA). Data are reported as the mean ± SD, unless otherwise noted. Body mass was analyzed using a matched pairs (pre- and post-treatment) *t*-test for each treatment group. Morphological data for individual NMJs were analyzed using a one-way ANOVA with the treatment group as a fixed effect. Based on previous data assessing the three-dimensional apposition of pre- and post-synaptic structures in 6-month-old mice [17], we estimated that four animals per treatment group would be sufficient to detect a 15–20% difference across treatment groups, with 80% power and α < 0.05. The study was not powered to detect a sex difference since previous studies have shown that there is no sex difference in diaphragm muscle cross-sectional area or function across ages in mice and rats [24,25]. The proportion of innervated, partially denervated, and fully denervated NMJs in each animal was analyzed using a two-way ANOVA with the treatment group and denervation category as fixed effects. Denervation category (innervated, partially denervated, or fully denervated) was evaluated using a Chi-square table and Pearson test. For each treatment group, the motor end-plate volume and relative planar area of individual NMJs were also analyzed across denervation categories using ANOVA. When appropriate, post hoc group differences were assessed using the Tukey–Kramer honestly significant difference test. A value of *p* < 0.05 was considered statistically significant.

## 3. Results

### 3.1. Chloroquine Treatment

To measure the effects of chloroquine on NMJ morphology, either chloroquine or vehicle was administered via intraperitoneal injection to adult male and female C57BL/6 × 129J mice for 7 days. As anticipated, no negative effects of chloroquine treatment on animal behavior or gross motor function were observed. The male mice were 41% heavier than the female mice, as expected (F_1,11_ = 43, *p* < 0.001; Table 1). There was no significant difference in body mass following 7 days of chloroquine treatment (t(5) = 1, *p* = 0.36) or vehicle treatment (t(5) = −1, *p* = 0.30).

### 3.2. Chloroquine Effects on Diaphragm Neuromuscular Junction Volume

Three-dimensional analyses of diaphragm muscle NMJ morphology were conducted using confocal imaging of en face fluorescently-labeled NMJs. Extensive labeling of motor end-plates using α-bungarotoxin was evident in all diaphragm muscle preparations. Labeling of pre-synaptic terminals and axons by immunoreactivity to synaptophysin was present in both treatment groups. In total, 491 individual diaphragm muscle NMJs were analyzed, with an average of 40 ± 11 NMJs per animal.

**Figure 1:**
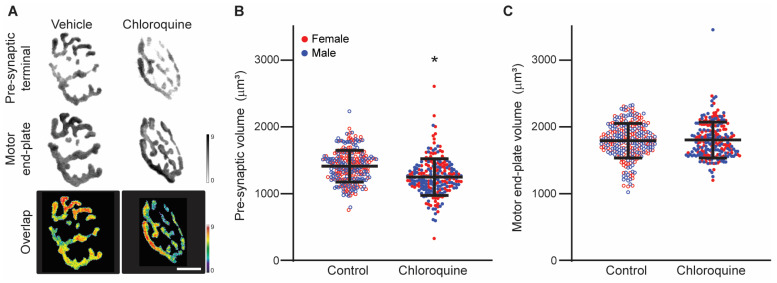
Pre-synaptic and motor end-plate volumes of diaphragm neuromuscular junctions (NMJs) from mice after 7 days of vehicle control or chloroquine treatment. (**A**) Representative images show 3D reconstructions of diaphragm NMJs from the vehicle and chloroquine treatment groups. Pre-synaptic terminals (labeled with synaptophysin) and motor end-plates (labeled with a-bungarotoxin) show varying depth by grayscale intensity (scale bar on the right; µm). The superimposed overlap images represent apposition of pre- and post-synaptic structures, with the depth shown on the pseudocolor scale bar on the right (µm; red–white reflects the greatest apposition). Scale bar: 10 µm. (**B**) Pre-synaptic terminal volume decreased by 12% with chloroquine treatment (1248 ± 274 µm^3^) compared to vehicle treatment (1411 ± 236 µm^3^). *, *p* < 0.05 effect of treatment. Lines and whiskers represent the mean ± SD. (**C**) There was no change in motor end-plate volume with chloroquine treatment (1804 ± 269 µm^3^) compared to vehicle treatment (1792 ± 259 µm^3^).

There was a significant effect of treatment on the pre-synaptic terminal volume (F_1,489_ = 50, *p* < 0.01; Figure 1), with the pre-synaptic terminal volume being 12% less in the chloroquine-treated group (1248 ± 274 µm^3^) compared to the vehicle-treated group (1411 ± 236 µm^3^). The volume of the motor end-plate did not change with treatment (F_1,489_ < 1, *p* = 0.63). The volume of the motor end-plate was 1804 ± 269 µm^3^ in the chloroquine-treated mice compared to 1792 ± 259 µm^3^ in the vehicle-treated group.

An index of NMJ complexity was obtained from the relative planar area occupied by the motor end-plate, as previously reported [17,18]. There was a significant effect of treatment on relative planar area (F_1,482_ = 4, *p* = 0.04). Relative planar area was ~3% less in the chloroquine-treated group (43 ± 7%) compared to the vehicle-treated group (45 ± 8% in the vehicle-treated group).

### 3.3. Morphological Assessment of Denervation of Diaphragm Neuromuscular Junctions

**Figure 2:**
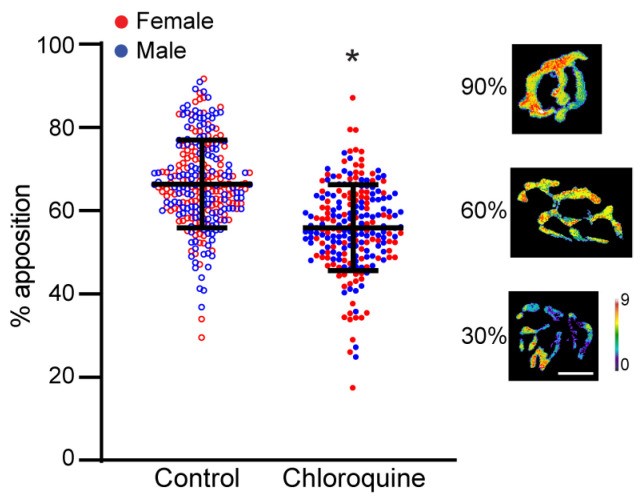
Three-dimensional apposition of pre- and post-synaptic structures of mouse diaphragm NMJs after 7 days of vehicle or chloroquine treatment. The percent apposition was calculated as the percent of the pre-synaptic terminal directly opposing the motor end-plate. There was a significant 15% relative change (decrease) in percent apposition in the chloroquine-treated mice when compared to the vehicle. *, *p* < 0.05 effect of treatment. Lines and whiskers represent the mean ± SD. The 3D reconstructions shown represent examples of NMJs with 90, 60, and 30% apposition of pre- and post-synaptic structures. Depth is shown on the pseudocolor scale bar on the right (µm; red–white reflects the greatest apposition). Scale bar: 10 µm.

Innervation of diaphragm NMJs was determined quantitatively through evaluation of the apposition of pre- and post-synaptic structures and semi-quantitatively using a visual classification of the overlap between pre- and post-synaptic structures. The percent of apposition was calculated for each NMJ as the fraction of the pre-synaptic terminal directly opposing the motor end-plate in three-dimensional analyses (Figure 2). There was a significant effect of treatment on the percent of apposition (F_1,489_ = 123, *p* < 0.01). Chloroquine treatment resulted in a significant 15% relative change (decrease) in percent of apposition (56 ± 10%) when compared to the vehicle treatment (66 ± 11%; *p* < 0.05).

**Figure 3:**
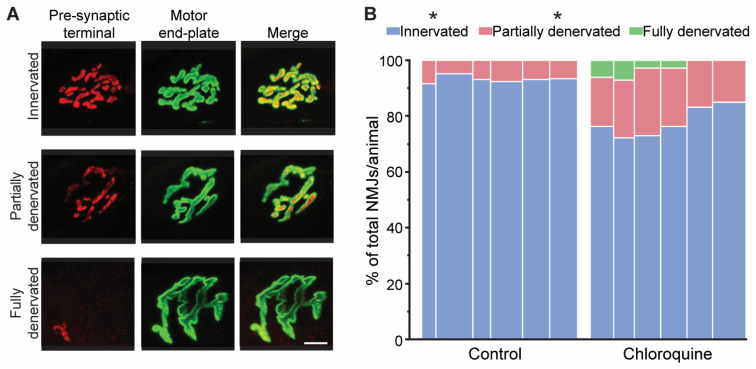
Diaphragm NMJ denervation in mice after 7 days of vehicle or chloroquine treatment. (**A**) Representative maximum intensity projection images of NMJs in each denervation category (i.e., innervated, partially denervated, or fully denervated). Full denervation was determined in NMJs that displayed minimal or no overlap between the pre- (red) and post-synaptic (green) structures based on maximum intensity projections of en face NMJs. Partial denervation was determined in NMJs that displayed partial overlap between the pre- and post-synaptic structures. Scale bar: 10 µm. (**B**) The proportion of NMJs in each denervation category was determined for each animal, and each individual animal is graphically represented above. The width of the bar represents the number of NMJs analyzed in that animal. Chloroquine treatment resulted in 16% fewer fully innervated NMJs than vehicle treatment (78 ± 5% compared to 93 ± 2%, respectively; *p* < 0.05). Chloroquine treatment increased the proportion of partially denervated NMJs by 2.7-fold compared to vehicle treatment (19 ± 3% compared to 7 ± 2%, respectively; *p* < 0.05). Fully denervated NMJs were only found in the chloroquine treatment group. * *p* < 0.05 effect of treatment.

All diaphragm NMJs were classified as being either innervated, partially denervated, or fully denervated based on the visual overlap between the pre- and post- synaptic structures on maximum intensity projections of en face NMJs. The proportion of NMJs in each denervation category (i.e., innervated, partially denervated, or fully denervated) was determined for each animal (Figure 3). The proportion of NMJs in each denervation category was significantly different (F_2,2_ = 2641, *p* < 0.01) with interaction between denervation category and treatment group (F_2,2_ = 63, *p* < 0.01). Post hoc analyses revealed that chloroquine treatment resulted in 16% fewer innervated NMJs than vehicle treatment (78 ± 5% compared to 93 ± 2%, respectively; *p* < 0.05). Chloroquine treatment increased the proportion of partially denervated NMJs by 2.7-fold compared to vehicle treatment (19 ± 3% compared to 7 ± 2%, respectively; *p* < 0.05). Finally, there was no significant effect of chloroquine treatment on the proportion of fully denervated NMJs, although six fully denervated NMJs were identified after chloroquine treatment and no fully denervated NMJs were found in the vehicle-treated animals.

### 3.4. Relationship Between NMJ Innervation and Morphology

**Figure 4:**
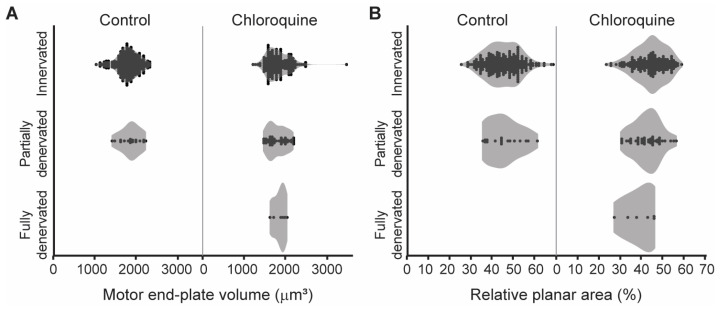
Violin plots of the post-synaptic motor end-plate volume and relative planar area of individual NMJs based on denervation category (innervated, partially denervated, or fully denervated). (**A**) There was no difference in motor end-plate volume across denervation categories in both the vehicle (F_1,260_ = 1, *p* = 0.28) and chloroquine groups (F_2,220_ < 1, *p* = 0.84). (**B**) There was no difference in relative planar area across denervation categories in both the vehicle (F_1,260_ < 1, *p* = 0.80) and chloroquine groups (F_2,220_ = 2, *p* = 0.10).

The post-synaptic motor end-plate volume and relative planar area of individual NMJs was also assessed based on denervation category (innervated, partially denervated, or fully denervated) for each treatment group to determine if the morphological characteristics were different in denervated versus innervated NMJs (Figure 4). There was no effect of denervation category on motor end-plate volume in both the vehicle (F_1,260_ = 1, *p* = 0.28) and chloroquine groups (F_2,220_ < 1, *p* = 0.84). The average motor end-plate volume was 1792 ± 269 µm^3^ in innervated NMJs (n = 417), 1804 ± 228 µm^3^ in partially denervated NMJs (n = 59), and 1839 ± 155 µm^3^ in fully denervated NMJs (n = 6). Similarly, there was no effect of denervation category on relative planar area in both the vehicle (F_1,260_ < 1, *p* = 0.80) and chloroquine groups (F_2,220_ = 2, *p* = 0.10). The average relative planar area was 45 ± 8% in innervated NMJs, 43 ± 7% in partially denervated NMJs, and 39 ± 7% in fully denervated NMJs. Taken together, these results suggest that the increased denervation observed following chloroquine treatment is not specific to NMJs of certain size or complexity.

## 4. Discussion

The present study examined diaphragm NMJ morphology and denervation following chloroquine treatment. Morphological changes were evident in the NMJs of chloroquine-treated compared to vehicle-treated mice. Chloroquine treatment resulted in a reduction in pre-synaptic volume and a subsequent decrease in the percent apposition of pre- and post-synaptic structures at diaphragm NMJs. Additionally, chloroquine treatment increased the proportion of partially and fully denervated NMJs. Notably, fully denervated NMJs were only found in chloroquine-treated animals. Since chloroquine is commonly used to inhibit autophagy by preventing autophagosome–lysosome fusion, these results highlight a potential role of autophagy in the maintenance of structural properties at adult NMJs in vivo.

### 4.1. Age-Related Effects on Neuromuscular Junction Morphology

Aging is known to cause morphological changes at the NMJ (c.f. [26,27]). The observed morphological change in decreased pre-synaptic volume without a change in post-synaptic volume in chloroquine-treated mice in the present study is similar to the age-related morphological changes previously reported in the diaphragm muscle NMJs of 18-month-old mice [17]. Pre-synaptic volume was ~20% less in 18-month-old mice compared to 6-month-old mice. Whereas the present study found a decrease in percent apposition of pre- and post-synaptic structures and an increase in the proportion of denervated NMJs after chloroquine treatment, there was no main effect of age on percent apposition or denervation in the previous study [17]. A recent study found that 12-month-old BALB/c mice had reduced diaphragm muscle motor end-plate area and no change in innervation (as assessed by the overlap between pre-synaptic proteins synapsin 1 and SNAP-25 to the motor end-plate) compared to 6-month-old mice [28]. Other studies have shown axonal swelling, motor end-plate fragmentation, and denervation in diaphragm NMJs in ~24-month-old mice [29] and rats [20].

### 4.2. Chloroquine Has Deleterious Effects on the Neuromuscular System

Chloroquine may contribute to neuromuscular dysfunction through morphological changes at the NMJ. Numerous prior studies have investigated the acute effects of chloroquine on neuromuscular transmission [5,30,31,32]. Ex vivo mouse studies in phrenic nerve–diaphragm preparations reported that ~45 min of chloroquine treatment at concentrations ≥ 5 µM decreased quantal release, suggesting an acute effect on pre-synaptic vesicle release and recycling [5]. Similarly, chloroquine at concentrations between 50 and100 µM resulted in decreased indirect muscle contraction elicited by supramaximal nerve stimulation in mouse, rat, and guinea pig diaphragm–phrenic nerve preparations within 21 to 35 min [30], as well as decreased frog rectus abdominis muscle contraction induced by acetylcholine or caffeine at chloroquine concentrations >10 µM [33]. Intraglossal injection of chloroquine at concentrations > 17 µM caused a decline in neuromuscular transmission by 12–35 min in frog gastrocnemius–soleus nerve–muscle preparations [31]. Chloroquine induced inhibition of voltage-gated pre-synaptic calcium currents in cortical rat neurons at 10 µM [32], but future work is necessary to explore the selectivity of chloroquine across motor neurons specifically. Regardless, chloroquine treatment has deleterious effects on neuromuscular transmission. Indeed, we recently reported that acute chloroquine treatment (50 mg/kg (~0.23 μM) via intraperitoneal injection) reduced maximum transdiaphragmatic pressure generation in old mice [8].

These prior studies examined the acute effects of chloroquine exposure whereas the present study consisted of a 7-day treatment duration (10 mg/kg/day). A previous study on diaphragm muscle found that chronic chloroquine treatment (45 mg/kg/day for 7–28 days) reduced twitch and tetanic forces [7]. Although chloroquine-induced myopathy that predominantly affects proximal limb muscles has been reported in individuals with a median age of 66 years old, it is reversible once chloroquine administration is discontinued [34]. Despite chloroquine‘s known side effect profile including neuromuscular effects, several studies have documented the safety and tolerability of chloroquine in long-standing clinical use across different age groups [2,35].

The chloroquine concentration in the present study is estimated to be ~0.6 μM for the 7-day treatment period by using chloroquine’s accumulation factor and assuming a single compartment pharmacokinetics model. This low dose of chloroquine appears to elicit effects primarily at the diaphragm NMJ pre-synaptic terminal, as evident by the observed decrease in pre-synaptic volume. Taken together, preclinical studies support the disruption of neuromuscular transmission, primarily at the pre-synaptic terminal, following chloroquine administration. The selectivity of chloroquine effects on the neuromuscular system is only partially understood.

### 4.3. Autophagy-Related Mechanisms of Chloroquine

One mechanism by which chloroquine acts is the inhibition of autophagy by preventing the fusion of the autophagosome with lysosomes [9]. Chloroquine has a strong propensity to concentrate in acidic lysosomes due to its properties as a weak base [36]. The amphiphilic nature of chloroquine allows for diffusion through organelle membranes; however, this only occurs at physiologic pH (7.4). After crossing the lysosomal membrane, chloroquine is protonated in the low pH environment, making it unable to travel back through the membrane. This results in chloroquine remaining inside the lysosome and subsequently interrupting the fusion of the autophagosome and the lysosome [9].

Intraperitoneal chloroquine injections at the same dosage as in the present study (10 mg/kg) resulted in the accumulation of mCherry-LC3 puncta in the myocardium by 4 h [16], increased LC3-II and LC3-II/LC3-I in mouse rectus femoris muscle after 8 weeks (five injections per week) [15], and increased LC3-II in the mouse liver after 2 weeks [37]. LC3-II protein levels were also increased at a lower chloroquine dosage (2.5 mg/kg) with a local injection in the epididymal adipose tissue of mice [38]. LC3 plays a complementary role with p62 in coordinating the degradation of cellular components. The same daily intraperitoneal chloroquine dosage (10 mg/kg) increases p62 protein levels in the mouse hippocampus after 8 weeks [39] and liver after 2 weeks [37]. Taken together, these changes in autophagy marker protein levels are consistent with an impairment of autophagy at the dose used in the present study and with this administration route.

Among the many pathologies associated with autophagy dysregulation, compromised autophagy has emerged as an effect of aging [40]. As organisms age, there is a notable decline in autophagic activity, leading to the accumulation of cellular waste and subsequent impaired function [41]. This decline in autophagy has been implicated in various age-related impairments, including diaphragm neuromuscular dysfunction [42]. Previous studies evaluating autophagy protein levels in phrenic and lumbar motor neurons found increased p62 and LC3 levels (indicative of autophagosome accumulation) in 24-month-old mice compared to 6-month-old mice [12,13]. These findings demonstrate neuromuscular autophagy impairment in motor neurons innervating the aged diaphragm muscle that may contribute to other neuromuscular hallmarks of aging in the diaphragm like the denervation of muscle fibers [17,24], subsequent sarcopenia [25,43,44], and motor neuron death [45]. The morphological changes observed were similar to those previously reported in the diaphragm muscles of 18-month-old mice [17]. These findings may implicate impaired autophagy as a mechanism of aging effects in diaphragm motor units. Indeed, acute chloroquine treatment decreased maximal transdiaphragmatic pressure generation in old mice, suggesting a susceptibility to autophagy impairments in old age [8].

One of the limitations of this study is that a single dose of chloroquine was used, and it is likely that the neuromuscular effects are dose dependent. In addition, the mice received chloroquine via intraperitoneal injection, which is expected to have systemic effects not limited to the diaphragm muscle. Indeed, chloroquine has immunomodulatory effects [36,46]; for example, human astroglial cells display pro-inflammatory responses through the activation of NF-kB after 100 mM chloroquine treatment [47]. Furthermore, despite recent use as an autophagy inhibitor, chloroquine likely exerts additional effects on cell processes other than autophagy. In nutrient-deficient endothelial cells, chloroquine (50 mM) acts via an oxidative stress-dependent mechanism to increase intracellular reactive oxygen species generation [48]. Chloroquine treatment (10 mM) also inhibits voltage-gated potassium channels (such as Kv1.3 channels) in T lymphocytes and reduces pro-inflammatory cytokine production [49]. Chloroquine’s anti-cancer effects are likely independent of autophagy and include a reduction in tumor growth in mouse models of melanoma after daily intraperitoneal injections (50–100 mg/kg/day) for 20 days [50]. While it is possible that the effect of chloroquine on NMJ morphology and denervation is not due to autophagy inhibition, the doses used in past studies were considerably higher or repeated for longer than those in the present study, and these differences may account for the more extensive side effect profile. Based on the results of the present study, there are important neuromuscular effects of autophagy inhibition using chloroquine, but the mechanism by which chloroquine causes diaphragm muscle denervation in mice should be investigated in future studies.

### 4.4. Autophagy Inhibition and Muscle Denervation

Prior reports document the effects of autophagy inhibition on axonal trafficking and suggest an important role in muscle denervation [51]. Autophagy inhibition with bafilomycin impairs axonal retrograde transport, a critical process for autophagosomes to reach lysosomes concentrated in the soma [52]. In agreement with this, autophagy inhibition results in autophagosomes accumulating predominantly in the distal axon and pre-synaptic terminals [53]. This accumulation of abnormal proteins can lead to oxidative stress [54] as well as the disruption of neural function and subsequent neurodegeneration [55].

A leading theory of the mechanism underlying age-related muscle denervation is oxidative stress, characterized by the overproduction of reactive oxygen species (ROS) [56]. Previous studies demonstrate that mice lacking the SOD1 enzyme have elevated oxidative stress and display muscle weakness, denervation, and sarcopenia [57]. Concurrently, autophagy is activated in response to oxidative stress, mitigating the impact by removing ROS-generating components [58]. However, when autophagy is impaired, there is an accumulation of toxic proteins and, thus, increased oxidative stress [59]. The interplay between oxidative stress and autophagy inhibition, where oxidative stress increases due to impaired autophagy, likely accelerates the denervation process. The autophagy-inhibitory effects of chloroquine may lead to increased oxidative stress, resulting in the observed denervation. A study in rats showed that treatment with hydroxychloroquine increased levels of the oxidative stress markers NO and MDA in the sciatic nerve and gluteal muscle [60]. These reports are consistent with our findings of chloroquine treatment resulting in denervation due to reduced pre-synaptic volume.

## 5. Conclusions

The present study highlights changes in diaphragm NMJ morphology induced by chloroquine treatment, namely a reduction in the three-dimensional apposition of pre- and post-synaptic structures and an increased proportion of denervated NMJs. Future studies will be important in determining whether disruptions in autophagy, the main mechanism of action of chloroquine, constitute an important mechanism underlying age-related neuromuscular dysfunction.

## Figures and Tables

**Table 1:** Characteristics of the experimental mice. The body mass of C57BL/6 × 129J mice (6 mice per treatment group, equal males and females) was measured at the start and end of a 7-day intraperitoneal injection treatment period with either vehicle or chloroquine (10 mg/kg/day). The male mice were 41% heavier than the female mice (F_1,11_ = 43, *p* < 0.001). There was no significant difference in body mass following 7 days of chloroquine treatment (t(5) = 1, *p* = 0.36) or vehicle treatment (t(5) = −1, *p* = 0.30). Values are the mean ± SD.

	Vehicle		Chloroquine	
	Male	Female	Male	Female
NMJs analyzed	136	134	106	109
Age (mo)	7.7 ± 0.6	6.7 ± 1.5	6.7 ± 0.6	7.3 ± 0.6
Pre-treatment body mass (g)	33.3 ± 2.9	22.6 ± 1.2	30.8 ± 3.4	22.8 ± 2.1
Post-treatment body mass (g)	32.3 ± 3.6	22.6 ± 0.9	30.6 ± 3.0	23.8 ± 2.1

## Data Availability

The data supporting this manuscript can be found within the text. Any additional data and the data that support the figures presented in this manuscript are available from the corresponding author upon reasonable request.

## Abbreviations

The following abbreviation is used in this manuscript:

NMJ	Neuromuscular junction
